# Risk factors associated with a breast cancer in a population of Moroccan women whose age is less than 40 years: a case control study

**DOI:** 10.11604/pamj.2016.24.19.8784

**Published:** 2016-05-06

**Authors:** Fatima Zahra Laamiri, Nadia Hasswane, Aicha Kerbach, Hassan Aguenaou, Youness Taboz, Hassna Benkirane, Mustapha Mrabet, Barkat Amina

**Affiliations:** 1 Faculty of Medicine and Pharmacy/Research Team in Maternal and Child Health and Nutrition, Mohammed V University, Rabat, Morocco; 2 Mixed Research Unit in Nutrition and Food URAC 39, (University Ibn Tufail-CNESTEN) Designated Regional Center of Nutrition Associate AFRA/IAEA, Kenitra, Morocco; 3 Laboratory of Hygiene and Community Medicine, Military Hospital of instruction Mohamed V in Rabat, Rabat, Morocco

**Keywords:** Breast cancer, risk factor, case-control study

## Abstract

**Introduction:**

Breast cancer is the most common cancer in morocco women were it occupies the first place in term of incidence and mortality. The aim of the present paper is to evaluate the risk factors associated with a breast cancer in a population of Moroccan women.

**Methods:**

A case-control study was conducted with population women whose age is less than 40 years during 2008-2010 at the National Institute of Oncology of Rabat. These women were interviewed for Epidemiological information and risk factor for breast cancer.

**Results:**

Included in this study were 124 cases and 148 age matched controls. No statistically significant case-control difference was found for the early age of menarche (OR = 2.474; CI 95%: 1.354- 4.521), and family antecedents of first degree of breast cancer (OR = 11.556; 95% CI: 2.548-52.411). However physical activity (OR = 0.507; 95% CI: 0.339 -0.757) early maternity age (OR = 0.212; 95% CI: 0.087 - 0.514), multiparity (OR = 0.742; 95% CI: 0.359 -1.539) and breastfeeding than 6 months (OR = 0.739; 95% CI: 0.357 -1.523) appear as significant protective factors.

**Conclusion:**

This study show the criminalization of only part of the known risk factors of breast cancer in this age group and confirms the probable protective role of physical activity and factors related to life reproductive women in our study (early childbearing, multiparity and lactation).

## Introduction

Breast cancer is a clinically heterogeneous and complex pathology [1] which occupies the first place in women in terms of incidence and mortality in the world [2, 3]. Therefore, it constitutes a major concern of public health, in the developed countries where it represents the first feminine cancer, and in developing countries [4]. Breast cancer is characterized by its frequency, its severity, its uneven geographical distribution and the increase of its impact across the world [5, 6]. In Morocco the breast cancer became the first feminine cancer. In 2005, the register of Rabat has registered 127 new cancer cases among women, 33.5% of female cancers. The incidence of breast cancer in Morocco is relatively higher than the other Maghreb countries but remains clearly lower than the Western countries where incidence rates are higher than 80 per 100,000 people [7]. In the etiology of breast cancer data on risk factors such as: age (the increase of the incidence after 30 years), body mass index (the risk increases after menopause), the family history related to BRCA1 or BRCA2 (5% of all cancers), hormonal factors (early menarche before age 12, late menopause after 55 years, no pregnancy or late pregnancies after age 30, oral contraceptives, lack of breastfeeding), family history of breast cancer, exposure to radiation, and hormone replacement therapy for menopause are very old. The epidemiological, clinical and experimental studies have allowed the collection of several information concerning the impact of these factors on the etiology of this cancer. But the results of these studies are controversial and vary considerably from one population to another and depending on the age For this reason, it seemed interesting to do a case-control study in a population of breast cancer Moroccan women whose age is less than forty years in order to make a descriptive analysis of the prevalence of risk factors related to this disease in this age group.

## Methods

**Recruitment of cases and controls**: We conducted a case-control study at the National Institute of Oncology Sidi Mohamed Ben Abdallah, Rabat in which we included a population of Moroccan women (collected between December 2008 and December 2010) we have divided into two groups, a group of women with breast cancer, and a group of breast cancer-free controls.

**Inclusion criteria:** The recruitment of breast cancer case has concerned a population of premenopausal women, newly diagnosed and whose age is less 40 years. The diagnosis of breast cancer was confirmed by mammography, biopsy and / or surgery by specialists doctors of the National Oncology Institute. Controls recruited at the same Institute within the framework of the cancer screening campaign organized by government authorities after having undergone a mammography that showed no signs of breast cancer. The age of these women must be less than 40 years.

**Exemption criteria**: For a group of women with breast cancer we excluded from the study: patients who were prescribed a treatment (chemotherapy, radiation therapy, hormone therapy); patients whose age is superior to 40 years; Postmenopausal women.

For a group of breast cancer-free controls: women whose age is superior than 40 years; postmenopausal women.

**Data collection**: The information was gathered from a basic questionnaire that has been evaluated and tested by our research team. The information collected concerned many risk factors implicated in mammary carcinogenesis and having been the subject of several epidemiological studies. The risk factors identified were: (a) age, (b) The body mass index (BMI), (c) Information on physical activity (d) The age at menarche, (e) age at first full-term pregnancy (f) the number of children; (g) the duration of breastfeeding for each child (h) oral contraceptives, (i) The hormone replacement therapy and finally (j) exposure to irradiation.

**Statistical analysis**: Risk factors collected in survey forms were carried out using the Statistical Package for Social Sciences (SPSS) 13.0 software. For bivariate analysis, the comparison of the means for quantitative and qualitative variables was carried out using Student's t-test and Pearson's Chi-square test, respectively. The results were expressed as mean ± standard deviation or in count and percentage. To identify susceptible and protective factors associated with breast cancer, comparisons between groups (women with breast cancer and breast cancer-free controls) were conducted using univariate logistic regression. The first level of significance was 5% and there was a confidence interval of 95%.

**Ethical considerations:** The administration of the National Institute of Oncology Sidi Mohamed Ben Abdallah, Rabat agrees to carry out the study for epidemiological purposes. Respect for the anonymity and confidentiality of information were strictly adhered to. Informed consent was signed before the inclusion of women in the study.

## Results

Two hundred and seventy two (272) women were included in our study and were divided into two populations, a population of one hundred and twenty four (124) women with breast cancer and a population of one hundred and forty eight (148) women who were breast cancer-free. Analysis of Table 1 shows that physical inactivity was statistically higher in the patient population than in the control group (22.6% versus 13.5%, P <0.001). However no statistically significant differences were observed for body mass index. The distribution of patients according to The hormonal risk factors (Table 2) shows that the percentage of women with early age of periods (below 12 years) was significantly higher in the patient population compared with controls (29% of cases versus 14.2% of controls; P = 0.03). However, there was no statistically significant difference between the two groups with respect to oral contraceptive and hormone replacement therapy. The distribution of patients according to the risk factors related to reproductive life (Table 3) shows a statistically significant difference between the two groups of women about the age of first pregnancy to term, the number of children and the average duration of breastfeeding. In deed the frequency of women with older age at the first full-term pregnancy (=30 years), the frequency of nulliparous women, and finally frequency of women who never breastfed their children were higher in the patient population as compared with controls (13,7% versus 6,1%, P <0,001;53.2% versus 25.7%, P < 0.001;54% versus 25%, P < 0.001 respectively). Regarding the genetic and environmental factors (Table 4) we found a statistically significant increase of women with a family history of breast cancer in particular the first degree. No significant association for breast cancer was found for exposure to radiation and family history of breast cancer among second degree relatives. In univariate analysis (Table 5) we found that the risk of breast cancer increases significantly with early menarche age [odds ratio (OR) = 2.474 confidence interval (CI) 95%: 1.354 - 4.521] and family history of first degree [OR = 11.556, CI 95%: 2.548 -52.411]. In contrast physical activity [OR = 0,507 CI 95% 0.339 – 0.757], early age [OR = 0,212 CI 95%: 0.087 -0.514], multiparity [OR = 0,742 CI 95% 0.359 - 1.539] and higher breastfeeding at 6 months [OR = 0,739 CI 95%: 0.357 - 1.523] were negatively associated with breast cancer. Regarding the body mass index, use of oral contraceptives, hormone replacement, family history of breast cancer among second degree and radiation exposure they have not been identified as predictive risk of breast cancer in this study.

**Table 1:** Demographic and epidemiological characteristics of control and breast cancer patients

	Control patients	Breast cancer patients	P-value
Characteristics	n= 148(%)	n = 124(%)	
Age group (mean ± SD years)	33,22±5,67	32,84±5,48	0,578
Physical activity			
**Low(Sedentary)**	20(13,5)	28(22,6)	0,002
**Moderate**	84(56,8)	80(64,5)	
**Hight**	44(29,7)	16(12,9)	
Body mass index (Kg /m^2^)	25,08±4,16	24,99±3,85	0,850
Body mass index (BMI) Group			
**Low**	8(5,4)	8(6,5)	0,482
**Moderate**	72(48,65)	64(51,6)	
**Overweight**	52(35,14)	40(32,3)	
**Obese**	16(10 ,81)	12(9,7)	

Notes: Values are expressed as mean ± standard deviation or in count and percentage. For body mass index (BMI) groups: low BMI = <18.5 kg/m^2^; normal BMI = 18.5 to <25 kg/m^2^; overweight BMI = 25 to <30 kg/m^2^; obese BMI = >30 kg/m^2^

**Table 2:** Hormonal factors in the groups of control and breast cancer patients

Characteristics	Control patientsn = 148(%)	Breast cancer patients n = 124(%)	*P-value*
Age menarche (mean ± SD years)	12,48±1,13	12,08±1,58	0 ,004
Menarche			
**< 12years**	21(14,2)	36(29)	0 ,03
**>ou = 12years**	127(85,8)	88(71)	
Oral contraceptifs			
**Never**	50(33,8)	66(53,2)	0,05
**<6 months**	8,5(57,4)	48(38,7)	
**≥6 months**	13(8,8)	10(8,1)	
Hormone replacement thérapie			
**No**	148(100)	124(100)	
**Yes**	0(0)	0(0)	

**Notes:** Values are expressed as mean ± standard deviation or in count and percentage

**Table 3:** Reproductive factors in the groups of control and breast cancer patients

Characteristics	Control patientsn = 148(%)	Breast cancer patients n = 124(%)	*P-value*
**Age at first full term pregnancy**			
**No**	39(26,4)	67(54)	<0,001
**<30years**	100(67,6)	40(32, 3)	
**≥30 years**	9 (6,1)	17(13,7)	
**Number of children (mean ± SD years)**	1,26±0,84	0,82±0,13	<0,001
**Number of birth**			
**Nulliparous**	38(25,7)	66(53,2)	<0,001
**1 to 2**	33(22,3)	14(11,3)	
**>3**	77(52)	44(35,3)	
**Duration of breastfeeding**			
**No**	37(25)	67(54)	<0,001
**<6months**	34(23)	14(11,3)	
**≥6 months**	77(52)	43(34 ,7)	

Notes: Values are expressed as mean ± standard deviation or in count and percentage

**Table 4:** Family history and health factors in the groups of control and breast cancer patients

Characteristics	Control patientsn = 148(%)	Breast cancer patientsn = 124(%)	*P- value*
Family historyof breast cancer			
**No**	148(100%)	115(92,7%)	0,001
**Yes**	0(0%)	9(7,3%)	
Breast cancer among degree relatives			
**First degree**	0(0%)	7(5,6%)	0,04
**Second degree**	0(0%)	2(1,6%)	
Exposure to irradiation			
**No**	143(96,6%)	120(96,8%)	0,944
**Yes**	5(3,4%)	4(3,2%)	

**Notes:** Values are expressed as mean ± standard deviation or in count and percentage

**Table 5:** Factors related to breast cancer analyzed by univariate analysis

Variables	OR^a^	95% CI	P -value
**Body mass index**	0,994	0,937 -1,05	0,849
**Physical activity**	0,507	0,339-0,757	0,001
**Early age at menarche (<12 years)**	2,474	1,354 -4,521	0,03
**Oral contraceptive (>6 months)**	1,716	0,696-4,232	0,005
**Early childbearing (<30 years)**	0,212	0,087-0,514	0,001
**Multiparity**	0,742	0,359-1,539	<0,001
**Lactation (>6 months)**	0,737	0,357-1,523	<0,001
**Family history of breast First degree**	11,556	2,548-52,411	0,002
**Second degree**	0,841	0,319-2,217	0,726
**Exposure to irradiation**	0,953	0,250-3,630	0,944

a Odds ratio (OR) has been adjusted for age by univariate logistic regression. Significance threshold *P* < 0.05.CI : confidence interval

## Discussion

This study realised in Morocco helped to highlight a set of risk factors related to carcinogenesis in a population of Moroccan women with breast cancer and whose age is less than forty years, namely the lifestyle, hormonal factors, factors related to genetics and reproductive life. The first part of this study is oriented towards the study of factors related to lifestyle. Our indicators were corpulence, physical activity and sedentary behavior of patients, and in this context we found no statistically significant association between BMI and the risk of breast cancer (Table 5). Indeed, the percentage of women having a weight and obese overload was similar in cases and controls in our population (Table 1). In literature obesity appears to be a significant risk factor after menopause. Thus women who are overweight more than 20 kg from the age of 18 have postmenopausal breast cancer risk doubled [8]. A relationship was found in the Nurses′ Health Study in postmenopausal women who never used hormone replacement [9]. At menopause, women who did not use estrogen and whose weight gain was 20 kg had a relative risk (RR) of 1.93 (95% CI: 1.43 to 2.73) [10]. In other studies the weight gain from the age of 40 years plays an important role in breast cancer etiology, increasing by 1.96 times and 2.5 the risk of this cancer respectively [11]. Taking into account the findings of these studies, we can make a connection with our results, showing both that women in our study were premenopausal and had never used hormone replacement. The Excess of adipose tissue leads to the increase of the production and the exposure to steroid hormones time [12]. Adipose tissue is also a storage site and metabolism of sex steroids. After menopause, the aromatization of androgens in the adipose tissue is one of the most significant sources of circulating estrogens. The results of this study also reveal a prevalence of sedentary lifestyle that proves highly significant among cases (Table 1). In contrast, high physical activity (PA) proves highly significant in contols. Physical activity seems to be considered as a protective factor according to the odds ratio obtained (Table 5). These results are supported by various epidemiological studies that focus on the importance of physical activity in the prevention of cancers including breast cancer [13]. In effect moderate physical activity (30 to 60 minutes least 4 times a week) reduces the risk of breast cancer by about 35%, particularly in postmenopausal women [14]. Physical activity is now a breast cancer protective factor proved, as well as a reduction factor in mortality from breast cancer [15]. The second part of this work focuses on the study of prevalence in this age group of the other risk factors implicated in breast carcinogenesis such as hormonal factors, genetic factors and factors related to reproductive life. Among hormonal factors we found that early age of menarche was associated significantly to the risk of breast cancer. The analysis of global data of our population shows that the percentage of women who menstruated before age 12 was significantly higher in cases compared to controls (29% versus14, 2%; P = 0.03) Table 2. This is consistent with data from a similar study which showed a significant increase in the percentage of women who got their first period at an early age only to the portion of age 22-34years [16]. This data is interesting in practice; early and targeted screening for breast cancer should be offered to our young women with early menarche history. In univariate analysis (Table 5) we found that early menstruation age increases of 24, 74% the risk of breast cancer [95% CI: 1.354 - 4.521] and this increase was statistically significant (P = 0.03). This is consistent with the literature that considers the early age of menstruation is associated to a risk of breast cancer [17]. So many studies have shown that women who got their first period between 14 to 15 years have 54% of breast cancer reduction compared with women who began menstruating aged < 12 years [18]. The biological basis of this association based on early and prolonged exposure to the hormonal impregnation that exists during the period of activity of the ovaries [19]. This hypothesis is consistent with high estrogen levels after menstruation, which is observed in women who have had their periods early [20]. Also no association was observed between breast cancer and taking oral contraceptive in our study (Table 5). In young women of childbearing age using oral contraceptives doesn't lead to the increase of risk, against the extensive use of contraceptives at a late age of reproductive life leads to a significant number of cases [21]. In this study the percentage of women using oral contraceptives more than 6 months was similar in both groups of women (Table 5). Meanwhile, the distribution of cases and controls according to factors related to reproduction (Table 3) allowed demonstrating a protective effect of early age at first birth, the multiparity and breastfeeding. In this study 53.2% of cases were nulliparous and 54% never breastfed their children. Unlike in the control population only 25.7% of women were nulliparous and 25% never breastfed their children.

In univariate analysis (Table 5) we found that multiparoty decreases of 74.2%the risk of breast cancer [95% CI: 0.359-1.539] and this decrease was statistically significant (P < 0.001). This is consistent with data literature which considers multiparity as a protective factor in mammary carcinogenesis. Indeed, after a transient increase in risk associated with pregnancy in the five years that follow it [22], the protective effect of long-term pregnancy exist [23]. Several mechanisms by which multiparity affects the risk of breast cancer are known or suspected. The reproductive period seems to have a double effect: the risk is increased immediately after delivery [24] and then gradually decreases. Pregnancy causes accelerated differentiation of breast tissue and rapid proliferation of the epithelium. The changes initiated during the first pregnancy, particularly if it occurred early are accented by each subsequent pregnancy, and breast cancer development is related to the rate of proliferation of mammary epithelial cells and inversely with the degree of differentiation [25]. The protective role of parity increases proportionally with the number of children and young age of first birth. Thus, a first pregnancy before age 30 reduces the risk by 25% compared to a woman who has no children [26]. In this study, the percentage of women having their first full-term pregnancy at an early age (below 30 years) was significantly decreased in cases compared to controls (32.3% vs 67, 6% Table 3). Furthermore we found that early maternal age (Table 5) decreased from 21.2% the risk of breast cancer [95% CI: 0.087; 0.514] and this decrease was statistically significant (P = 0.001). Thus women having their first full term pregnancy before age 30 are at risk of breast cancer decreased by 25% compared to nulliparous women [27]. The protective effect of multiparity appears to increase as the number of deliveries [24]. In addition we found that the percentage of women whose average duration of breastfeeding is more than six months was statistically decreased in cases compared with control women (34.7% vs 52%, Table 3) and this reduction was statistically significant (P <0.001). Analysis of global data has highlighted an inverse association between duration of breastfeeding and risk of breast cancer [OR = 0.652 95% CI: 0.546; 0.780]. Breastfeeding as a protective factor has been the subject of several studies and the results are controversies. According to studies the risk of breast cancer is reduced by more than 4% for each 12-month lactation period [28], and this reduction in risk is higher in young women than in older women [20]. Thus the protective effect of breastfeeding increases with increasing duration of breastfeeding.

This inverse association between breastfeeding and the risk of breast cancer may be explained by the following biological mechanisms: by a reduction in estrogen and an increase in the production of prolactin, which are supposed to reduce cumulative exposure to estrogen in women [20]; Other hand has been shown that the level of estrogen in the blood of nursing women gradually increases from the last birth and continues for several years before reaching the level that is recorded in nulliparous women [29]; The pH of the milk from the breasts of women who have not breastfed are significantly elevated in comparison with that from women′s breasts have already breastfed. During lactation milk is acidic. Epithelial cells in an alkaline environment, undergo alterations such as hyperplasia, atypia, and increased mitotic activity [30].

Finally, the protective effect of breastfeeding is due to its role in the shift of restoring ovulation. Meanwhile we have collected through the basic questionnaire information related to other risk factors such as exposure to radiation and genetic factors such as family history of first and second degree. We found a positive association between family history first degree and risk of breast cancer (Table 5). Family history exposed to breast cancer risk and this risk is even more important with the increasing number of first-degree history. An epidemiological study [28] helped to highlight an increased risk of 80% when there is a history in the first degree, three times if two first-degree history coexist and four times in the event of three or more history [28]. In this study we found a statistically significant increase in breast cancer with 1st degree among cases compared to controls (5.6% vs 0% Table 4). The overall odds ratio analysis shows that family history of first degree increase the breast cancer risk of 11, 556 [95% CI: 2.548; 52.411] Table 5. Regarding exposure to radiation and family history of breast cancer 2^nd^ degree (Table 5), they were not identified as risk factors for breast cancer in the study population. Given the encouraging results of this study, It would be interesting to confirm them by prospective studies especially those related to environmental factors in order to individualize populations where the risk is more important and may be possible to target the cause.

## Conclusion

This case-control type study has allowed us to make a descriptive analysis of the prevalence in a population of Moroccan women whose age is less than forty years, a number of risk factors implicated in mammary carcinogenesis and having been the subject of several epidemiological studies namely hormonal factors, genetic factors and factors related to reproductive life. The results obtained allowed to demonstrate the criminalization of only some of the known risk factors, in particular the early age of menarche and first degree family history of breast cancer. However, physical activity, early age at first full-term pregnancy, multiparity and lactation appear to be the most important protective factors in this study. These results reinforce the suspicion hormonal factors, factors related to reproduction and genetic factors in the incidence of breast cancer in Morocco. Their knowledge is essential for public health action.

### What is known about this topic

- Breast cancer is a complex and multifactorial disease. Several risk factors are known among which are essential factors such as age (the probability of breast cancer increases in a major way from 40 years), family history in particular the first degree. But also secondary factors that influence more or less significantly the risk of a particular breast cancer factors related to gynecologic obstetric history (early puberty, late menopause, first pregnancy after 30 years) and factors related to lifestyles such as physical activity.

### What this study adds

- The notion of risk factors for breast cancer is obsolete if we don't consider women's life period of the study;
- Engaging in regular physical activity is essential to reduce the risk of breast cancer in young women;
- In the framework of the national program of early detection of breast cancer, screening should be offered to young women with early menarche history.
